# Low CD4/CD8 T-Cell Ratio Associated with Inflammatory Arthropathy in Human T-Cell Leukemia Virus Type I Tax Transgenic Mice

**DOI:** 10.1371/journal.pone.0018518

**Published:** 2011-04-01

**Authors:** Takeo Ohsugi, Toshio Kumasaka

**Affiliations:** 1 Division of Microbiology and Genetics, Center for Animal Resources and Development, Institute of Resource Development and Analysis, Kumamoto University, Kumamoto, Japan; 2 Department of Pathology, Japanese Red Cross Medical Center, Tokyo, Japan; Facultad de Medicina, Uruguay

## Abstract

**Background:**

Human T-cell leukemia virus type I (HTLV-1) can cause an aggressive malignancy known as adult T-cell leukemia/lymphoma (ATL) as well as inflammatory diseases such as HTLV-1-associated myelopathy/tropical spastic paraparesis (HAM/TSP). A transgenic mouse that expresses HTLV-1 Tax also develops T-cell leukemia/lymphoma and an inflammatory arthropathy that resembles rheumatoid arthritis. The aim of this study was to identify the primary T-cell subsets involved in the development of arthropathy in Tax transgenic mice.

**Principal Findings:**

By 24 months of age, Tax transgenic mice developed severe arthropathy with a cumulative incidence of 22.8%. The pathological findings of arthropathy in Tax transgenic mice were similar to those seen in human rheumatoid arthritis or mouse models of rheumatoid arthritis, with synovial proliferation and a positive rheumatoid factor. Before the onset of spontaneous arthropathy, young and old Tax transgenic mice were not sensitive to collagen and did not develop arthritis after immunization with type II collagen. The arthropathic Tax transgenic mice showed a significantly decreased proportion of splenic CD4^+^ T cells, whereas the proportion of splenic CD8^+^ T cells was increased. Regulatory T cells (CD4^+^CD25^+^Foxp3^+^) were significantly decreased and CD8^+^ T cells that expressed the chemokine receptor CCR4 (CD8^+^CCR4^+^) were significantly increased in arthropathic Tax transgenic mice. The expression of *tax* mRNA was strong in the spleen and joints of arthropathic mice, with a 40-fold increase compared with healthy transgenic mice.

**Conclusions:**

Our findings reveal that Tax transgenic mice develop rheumatoid-like arthritis with proliferating synovial cells in the joints; however, the proportion of different splenic T-cell subsets in these mice was completely different from other commonly used animal models of rheumatoid arthritis. The crucial T-cell subsets in arthropathic Tax transgenic mice appear to resemble those in HAM/TSP patients rather than those in rheumatoid arthritis patients.

## Introduction

Human T-cell leukemia virus type I (HTLV-1) was the first human retrovirus to be isolated. Infection with HTLV-1 can result in an aggressive malignancy known as adult T-cell leukemia/lymphoma (ATL) or in inflammatory diseases, such as HTLV-1-associated myelopathy/tropical spastic paraparesis (HAM/TSP), after a prolonged period of latency often lasting between 20 and 50 years [1]. The lifetime incidence of ATL among carriers of HTLV-1 is estimated to be 1 to 5%, whereas that of HAM/TSP is 0.3 to 4% [2]. The lifetime incidence of HTLV-1-associated diseases in general, including ATL, HAM/TSP, and other inflammatory diseases such as uveitis, polymyositis, and arthropathy, may be close to 10% [2]. Why HTLV-1-infected individuals develop different types of diseases and the mechanisms through which HTLV-1 causes these diseases remain unclear.

The *tax* gene product encoded by the *pX* region of the HTLV-1 genome appears to be a key element in the development of HTLV-1-associated diseases [1]–[4]. Tax enhances productive virus replication by driving viral gene transcription through the cAMP-responsive element located in the long terminal repeat of the viral genome [3], [4]. Tax also activates the expression of many cellular genes, including those for cytokines, cytokine receptors, and immediate early transcription factors via cellular signal transduction pathways, such as those mediated by the transcription factor nuclear factor-kappaB (NF-κB) and serum response factor [3], [4].

One of the best ways to investigate the role of Tax in pathogenesis *in vivo* is to create a transgenic mouse model that expresses Tax and to study the model before and after disease develops. Accordingly, transgenic mouse models carrying *tax* have been shown to develop mesenchymal tumors, neurofibromas, arthropathy, and large granular lymphocytic leukemia; however, none of these transgenic mice develops a T-cell leukemia/lymphoma resembling ATL [5]. Recently, transgenic mice were created in which *tax* expression was restricted to thymocytes by using the proximal promoter of the lymphocyte-specific protein tyrosine kinase (*Lck*) gene. These mice developed pre-T-cell (CD4^–^CD8^–^) leukemia/lymphoma [6]. Furthermore, our group created a transgenic mouse model of HTLV-I by using the distal promoter of *Lck* to express *tax* in mature thymocytes and peripheral T lymphocytes. Our transgenic mice developed a mature T-cell leukemia/lymphoma (CD4^+^ or CD8^+^) similar to ATL [7]. Our results suggest that HTLV-1 Tax promotes oncogenesis not only in immature T cells but also in mature T cells.

While expanding our *Lck*-distal-*tax* transgenic mouse colony, we found that inflammatory arthropathy developed among the Tax-positive mice without leukemia but not among their Tax-negative littermates. The inflammatory disease developed after a prolonged latency period, like that seen among humans infected with HTLV-1. Inflammatory arthropathy has been reported for other Tax transgenic mouse models [8], [9]; however, leukemia does not also occur in these other models. Our Tax transgenic mouse model is the first to model both neoplastic and inflammatory disease, similar to HTLV-1 infection in humans.

Recent advances using conventional animal models of rheumatoid arthritis have led to a better understanding of the role of different immune cells in the pathogenesis of disease. Treatment with an antibody against CD4 suppresses the onset of rheumatoid arthritis, suggesting a role for CD4 T cells in disease progression [10]. CD4 T cells are classified into Th1, Th2, and Th17 subsets according to the type of cytokine produced [11]. Th1 cells, which also produce interferon-γ (IFN-γ), have traditionally been considered to be the major player in collagen-induced arthritis [12]; however, recent data suggest a central role for IL-17-producing Th17 cells in the pathogenesis observed in the collagen-induced arthritis model [13], [14]. In addition, CD4^+^CD25^+^Foxp3^+^ regulatory T (Treg) cells appear to have suppressive or regulatory activity and play a crucial role in maintaining self-tolerance [15]. The transfer of exogenous CD4^+^CD25^+^ T cells or Foxp3^+^-transduced CD4^+^ cells from healthy mice into pre-arthritic mice inhibits the development of arthritis, suggesting a regulatory role for Treg cells in the development of arthritis [16]–[18].

Little is known about the immune cell subsets that play important roles in the pathogenesis of arthritis induced by HTLV-1 Tax. Furthermore, it is unclear whether the inflammatory disease that develops in Tax transgenic mice is similar to that which develops in humans infected with HTLV-1. To address these issues, we identified the crucial lymphocyte subsets required for the development of arthropathy in Tax transgenic mice.

## Results

### Incidence of disease in Tax transgenic mice

**Figure 1:**
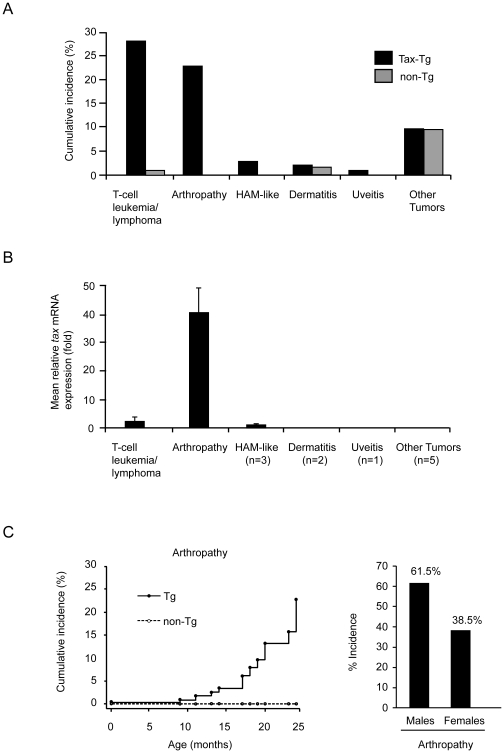
Incidence of diseases in Tax transgenic mice. Over a 2-year period, we studied 114 Tax transgenic (Tax-Tg) mice and 105 non-transgenic (non-Tg) mice. (A) Major phenotypes of disease were mature T-cell leukemia/lymphoma and arthropathy. The mice developed mature T-cell leukemia/lymphoma with an incidence of 28.1% and severe arthropathy with an incidence of 22.8%. (B) Expression of *tax* mRNA in lesions of arthropathic Tax transgenic mice relative to that in the spleens of healthy transgenic mice (n = 5 per group). Higher levels of *tax* mRNA were found in the joints of arthropathic Tax transgenic mice compared with leukemic Tax transgenic mice (n = 5 per group). (C) Arthropathy develops in Tax transgenic mice after a prolonged latency period of at least 9 months (left panel). The distribution of the cumulative incidence of arthropathy according to gender: male mice have an apparent higher tendency to develop arthropathy than female mice, although the difference was not statistically significant (right panel; *P* = 0.082, one-tailed Fisher's exact test).

We previously reported on the development of mature T-cell leukemia/lymphoma in Tax transgenic mice [7]. In the present study, we determined the 2-year cumulative incidence of other diseases in 114 Tax transgenic mice and 105 non-transgenic mice (Figure 1A), and we used real-time RT-PCR to quantify *tax* mRNA expression in samples from organ lesions of Tax transgenic mice and in splenocytes from transgenic mice free of disease (Figure 1B). The incidence of mature T-cell leukemia/lymphoma was 28.1% in Tax transgenic mice, whereas the incidence was only 1% in non-transgenic mice (Figure 1A). The leukemic cells in the spleens of leukemic mice exhibited a modest increase (0.3- to 8-fold) in *tax* mRNA expression compared with splenocytes from disease-free transgenic mice (Figure 1B). Severe arthropathy was absent in non-transgenic mice, but the incidence among Tax transgenic mice was 22.8% (Figure 1A). The joints of arthropathic Tax transgenic mice showed a 14.1- to 72.5-fold increase in *tax* mRNA expression compared with expression in the spleens of healthy transgenic mice (Figure 1B). None of the Tax transgenic mice developed both leukemia and arthropathy.

Three Tax transgenic mice developed HAM/TSP-like disease with symmetrical paraparesis of the hind legs (Figure 1A); however, the disease was not inflammatory, but involved a proliferation of lymphoid tumor cells in the spinal cord (data not shown). A few Tax transgenic and non-transgenic mice showed intense dermatitis, but *tax* mRNA expression was absent in the lesions of diseased Tax transgenic mice (Figure 1A, B). Other tumors observed in both groups of mice included B cell lymphoma, hepatocarcinoma, and ovarian cancer, but *tax* mRNA was not expressed in these tumors (Figure 1A, B).

We reported previously that the cumulative incidence of T-cell leukemia/lymphoma increases over time [7]. In our present study, we found that the cumulative incidence of arthropathy in Tax transgenic mice also increased over time (Figure 1C, left). Similar to the findings for T-cell leukemia/lymphoma, arthropathy occurred after a prolonged latency period of at least 9 months (Figure 1C, left). The arthropathic Tax transgenic mice showed erythema and swelling of the foot pads and marked swelling of the ankles and digits, but they did not show intense gait disturbance. There was no statistically significant gender difference in the 2-year cumulative incidence of arthropathy; however, there was an apparent trend that male mice had a somewhat higher cumulative incidence than female mice (Figure 1C, right; *P* = 0.082, one-tailed Fisher's exact test).

### Clinical signs of arthropathy in Tax transgenic mice

**Figure 2:**
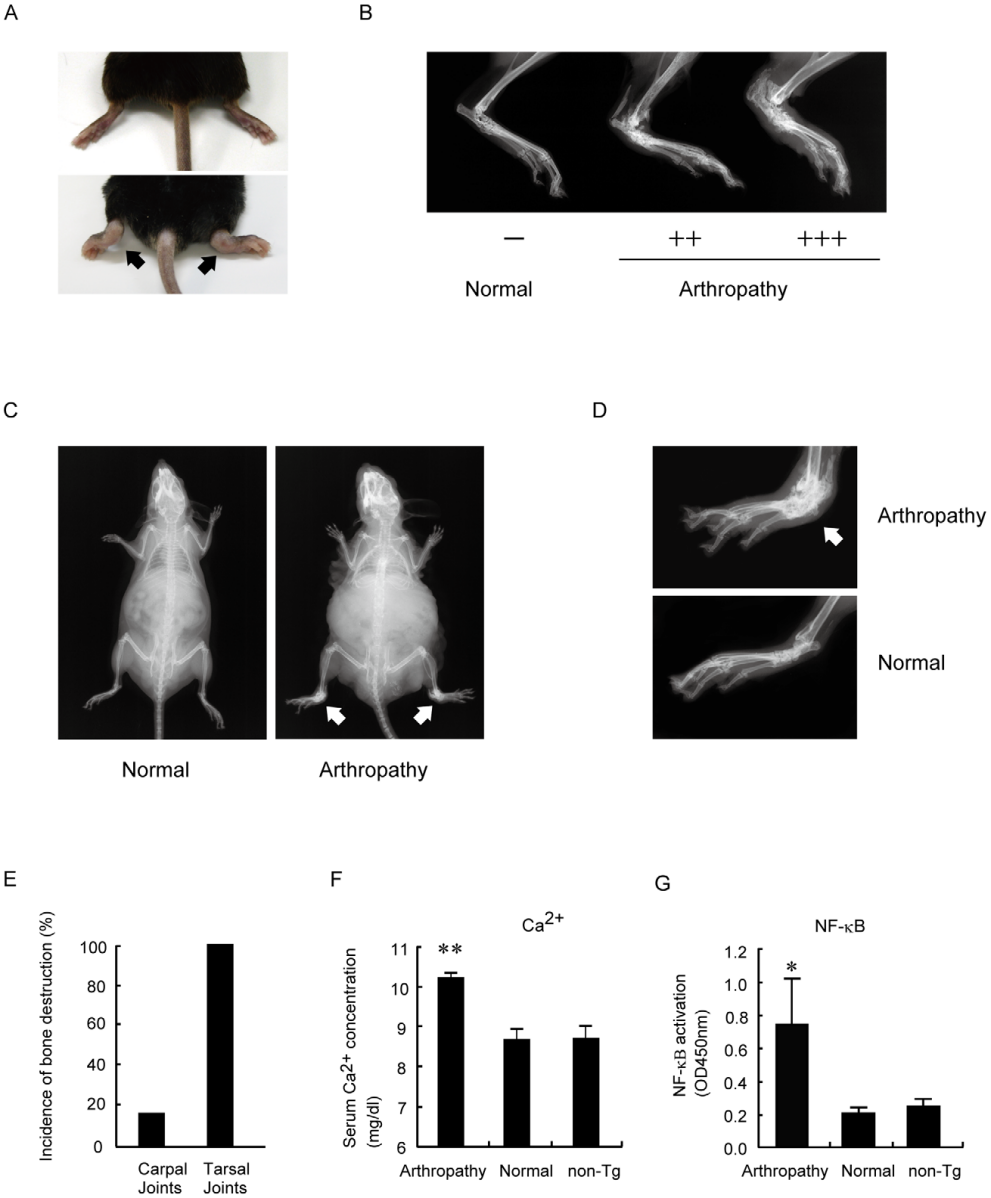
Arthropathy in Tax transgenic mice. (A) Hind legs of control non-transgenic mice (upper panel). Marked swelling of the ankles and digits of arthropathic Tax transgenic mice (lower panel). (B) Representative radiographic images of the hind legs of arthropathic Tax transgenic mice (Arthropathy) showed that the ankle joints were eroded, deformed, exhibited ankylosis, and clinically scored 2 (middle image) and 3 (right image). There was no lesion, however, in clinically healthy transgenic mice (Normal; left image). (C) Radiographic images of the whole body of a clinically healthy (left panel) mouse and an arthropathic (right panel) transgenic mouse. Bone destruction was observed mainly in the tarsal joint bilaterally (arrowheads). (D) Magnification of the radiographic images (4×) of the right hind legs in arthropathic (upper panel) and healthy (lower panel) transgenic mice. Bone destruction was restricted to the tarsal joint in arthropathic mice (arrow). (E) Bone destruction was observed in the tarsal joint bilaterally in all cases of arthropathic Tax transgenic mice. In some arthropathic Tax transgenic mice, bone destruction was also observed in the carpal joint bilaterally (16.6%, n = 24). (F) Serum calcium levels of arthropathic Tax transgenic mice (Arthropathy; n = 8) and age-matched healthy transgenic mice (Normal; n = 8) and non-transgenic control mice (non-Tg; n = 8). The serum calcium levels in arthropathic Tax transgenic mice were significantly increased compared with non-transgenic mice. ** *P*<0.01. (G) NF-κB activation in lesions of diseases in transgenic mice. Inflammatory cells from the joints of arthropathic Tax transgenic mice showed constitutive activation of NF-κB, which was significantly elevated compared with normal cells derived from healthy transgenic and non-transgenic control mice (n = 5 per group). * *P*<0.05.

The progression of arthropathy in Tax transgenic mice initially involved the hind limbs (Figure 2A, lower panel), whereas these symptoms were absent in healthy non-transgenic littermates (Figure 2A, upper panel). The radiographic images of the hind legs in arthropathic Tax transgenic mice showed that the ankle joints were eroded and deformed, and they exhibited ankylosis (Figure 2B); the clinical arthritis score was 0 (left) for a healthy transgenic mouse, and 2 (middle) or 3 (right) for an arthropathic Tax transgenic mouse. Furthermore, calcification of ligaments and spur formation were also observed in arthropathic Tax transgenic mice. In age-matched mice without Tax expression, the joints were always normal.

Whole-body radiographic images of arthropathic Tax transgenic mice showed mainly bone destruction of the tarsal joints bilaterally (Figure 2C, D). In some instances, bone destruction was also observed in the carpal joints (Figure 2E), and in rare instances bone destruction was observed in the knees (data not shown). Hypercalcemia is frequently observed in ATL patients [19]. Serum calcium levels in arthropathic transgenic mice were significantly higher compared with age-matched healthy transgenic and non-transgenic mice (Figure 2F).

NF-κB is a transcription factor, and its activation is increased in the synovium of patients with rheumatoid arthritis [20] and is thought to be implicated in the cartilage destruction that accompanies rheumatoid arthritis [21]. We measured the DNA-binding activity of NF-κB in the joints of arthropathic Tax transgenic mice using an ELISA. The inflammatory cells in the joints of arthropathic Tax transgenic mice had significantly higher levels of NF-κB compared with cells in the joints of healthy transgenic mice (Figure 2G). There was no difference in NF-κB activation between healthy transgenic mice and healthy non-transgenic mice from the same litter.

### Histological findings of arthropathy in Tax transgenic mice

**Figure 3:**
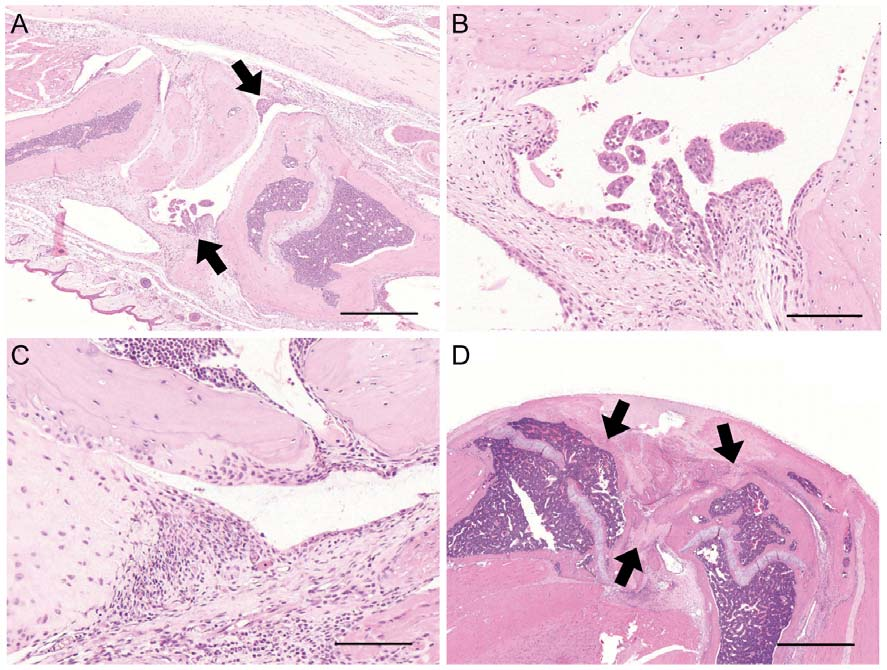
Histological findings of arthropathy in Tax transgenic mice. (A) An ankle joint showed papillary proliferation of synovial cells (arrowheads) and irregular surface of the joint (bar, 500 µm). (B) A high-power view showed lymphocyte infiltration and capillary proliferation at the area of synovial proliferation in panel A (bar, 100 µm). (C) Synovial proliferation and infiltrating inflammatory cells caused disappearance of cartilage of the articular surface and erosion of the bone similarly to pannus (bar, 100 µm). (D) A knee joint rarely showed severe destruction of the articular surface or fibrous adhesion of bones, indicated by black arrows, similarly to fibrous ankylosis (bar, 1000 µm).

Arthropathic Tax transgenic mice developed splenomegaly and lymphadenopathy; however, the arthropathic Tax transgenic mice showed no evidence of atypical lymphocytes, such as leukemic cells, in the spleen or lymph nodes (data not shown). The main histological finding of rheumatoid arthritis in humans is an inflamed synovium [22]. Marked proliferation of the synovial tissues was observed in arthropathic Tax transgenic mice, as shown in Figures 3A and B. The ankle joints of arthropathic Tax transgenic mice showed papillary proliferation of synovial cells (Figure 3A, arrows) and irregular surface of the joint. Synovial proliferation and intense infiltration with inflammatory cells was observed in the joints of arthropathic Tax transgenic mice. Furthermore, synovial pannus-like granulation tissues invaded bone and cartilage in arthropathic Tax transgenic mice (Figure 3C). The knee joints rarely showed severe destruction of the articular surface or fibrous adhesion of bones, similar to the fibrous ankylosis seen in rheumatoid arthritis patients (Figure 3D; arrows).

Augmentation of proinflammatory cytokine and autoantibody production in arthropathic Tax transgenic mice.

**Figure 4:**
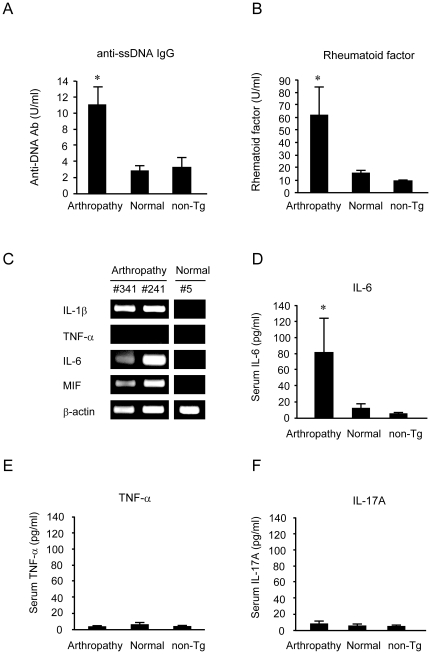
Level of autoantibodies and cytokine production in Tax transgenic mice. (A, B) Both anti-ssDNA and rheumatoid factor were elevated significantly in arthropathic Tax transgenic mice (Arthropathy; n = 7) compared with age-matched normal transgenic mice (Normal; n = 9) and non-transgenic mice (non-Tg; n = 9). (C) Cytokine mRNA expression in the joints of arthropathic and healthy Tax transgenic mice. Expression of mRNAs for the proinflammatory cytokines IL-1β, IL-6, and MIF was detected in arthropathic Tax transgenic mice but not in healthy transgenic mice. TNF-α expression, however, was not detected in either arthropathic or healthy transgenic mouse joint RNA. (D–F) Serum IL-6, TNF-α, and IL-17A concentrations in arthropathic (n = 15) and healthy (n = 11) transgenic mice, as well as that of age-matched non-transgenic control mice (n = 10). Serum IL-6 levels in arthropathic transgenic mice were significantly increased compared with non-transgenic mice. * *P*<0.05. There was no difference in levels of serum TNF-α or IL-17A between mouse groups.

We measured serum levels of rheumatoid factor and antibodies against single-stranded DNA (ssDNA) for evidence of autoimmunity. Both anti-ssDNA and rheumatoid factor were elevated significantly in arthropathic Tax transgenic mice compared with age-matched healthy Tax transgenic and non-transgenic mice (Figure 4A, B). We next used semi-quantitative RT-PCR to investigate cytokine gene expression in the joints of arthropathic Tax transgenic mice (Figure 4C). Expression of mRNAs encoding the proinflammatory cytokines interleukin-1β (IL-1β), interleukin-6 (IL-6), and macrophage migration inhibitory factor (MIF) was detected in the joints of arthropathic Tax transgenic mice but not in the joints of healthy transgenic mice from the same litter. Tumor necrosis factor-α (TNF-α) expression was not detected in the joints of either arthropathic or healthy transgenic mice.

We next used ELISA to measure serum levels of the proinflammatory cytokines IL-6, TNF-α, and interleukin-17A (IL-17A) in arthropathic mice. Serum IL-6 concentrations in arthropathic Tax transgenic mice were significantly higher than those in healthy transgenic mice or non-transgenic mice (Figure 4D). Serum levels of TNF-α and IL-17A were below the detection limits of the ELISAs we used (Figure 4E, F).

### Induction of arthritis in Tax transgenic mice with type II collagen

**Figure 5:**
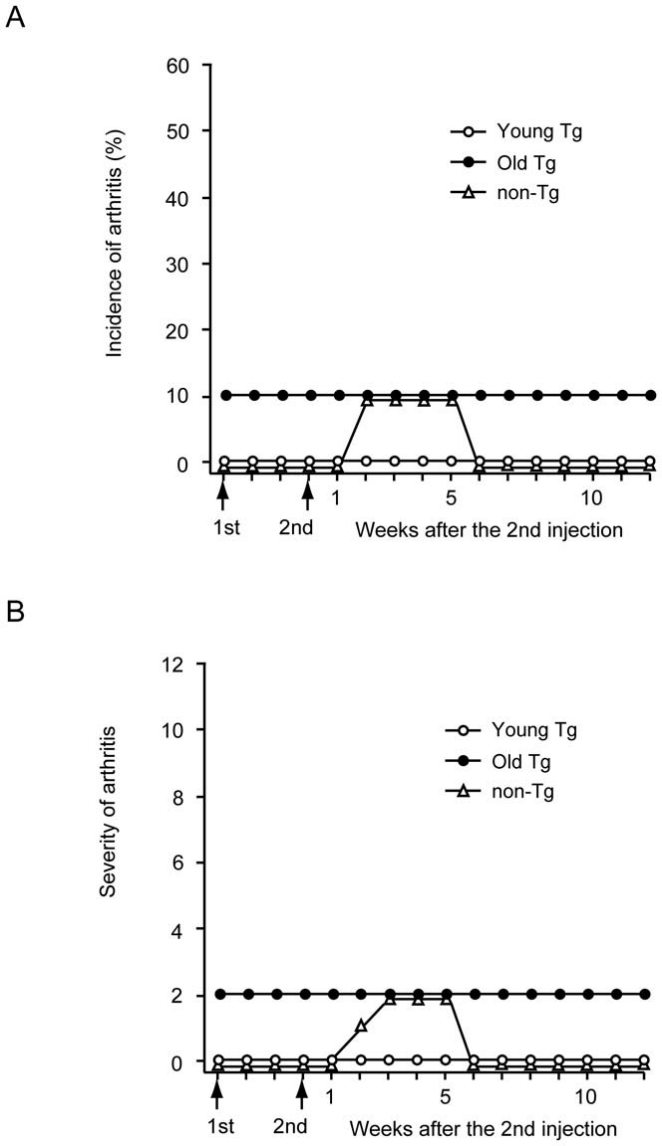
Incidence and severity of arthropathy in Tax transgenic mice injected with type II collagen. Young Tax transgenic mice (Young Tg; 2–3 months of age; ○), old Tax transgenic mice (Old Tg; 15–16 months of age; •) and non-transgenic mice (non-Tg; 2–3 months of age; Δ) were used. All mice were male (10 mice/group) and were immunized with type II collagen in complete Freund's adjuvant (day 0; 1st) and boosted with type II collagen in incomplete Freund's adjuvant at day 21 (2nd). One of the old Tax transgenic mice developed mild arthropathy before the first immunization. The incidence (panel A) and severity (panel B) of arthritis was determined. All three mouse groups were resistant to the development of type II collagen–induced arthritis.

Type II collagen can induce arthritis in rats and mice [23]. The major histocompatibility complex loci have been found to be important in influencing the susceptibility of mice to type II collagen. Mice with the DBA/1 (H-2^q^) or B10.RIII (H-2^r^) haplotype, and their congenic strains, are susceptible to collagen-induced arthritis [24]–[26]. Mice without the H-2^q^ or H-2^r^ haplotypes rarely develop collagen-induced arthritis. Some studies, however, have reported that *pX* transgenic mice (H-2^k^) injected with type II collagen develop collagen-induced arthritis [27], [28]. Therefore, we examined the susceptibility of Tax transgenic mice of the strain DBA/2 (H-2^d^) × C57BL/6 (H-2^b^) to type II collagen–induced arthritis. In contrast to earlier reports [27], [28], none of the 10 transgenic mice developed arthritis (Figure 5A). Because of the long latency period until the development of arthropathy in our Tax transgenic mice, we also examined the susceptibility of old Tax transgenic mice to type II collagen–induced arthritis. One of 10 old Tax transgenic mice (10%) developed weak arthropathy (total arthritis score 2) before inoculation with type II collagen; however, the arthropathy observed in that mouse did not become more severe after inoculation with type II collagen, and none of the other mice developed arthropathy (Figure 5B).

### Percentages of CD4^+^ T cells and CD8^+^ T cells in splenocytes from arthropathic Tax transgenic mice

**Figure 6:**
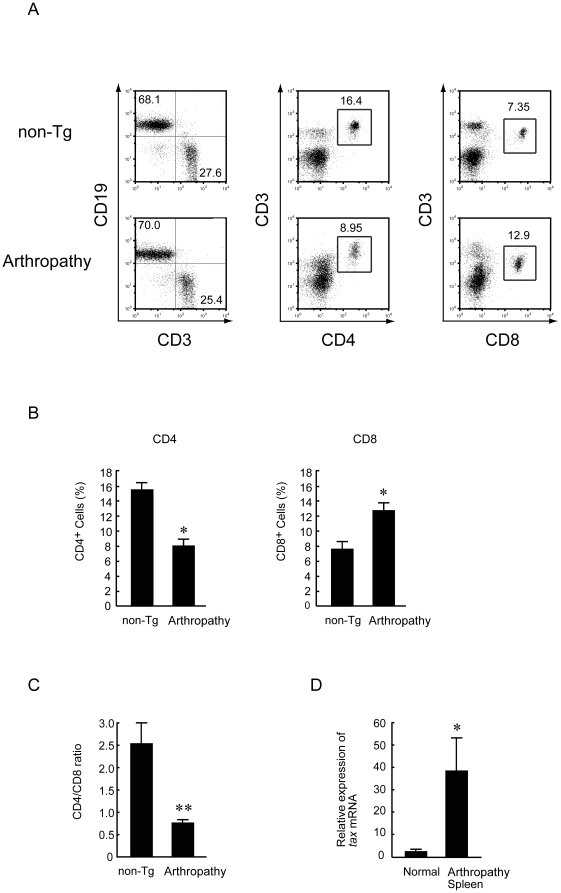
Phenotype of T cells in arthropathic mice. (A) A representative flow cytometric analysis of splenocytes stained with monoclonal antibodies to examine the expression of CD19 versus CD3, CD3 versus CD4, and CD3 versus CD8 in arthropathic Tax transgenic mice (Arthropathy; lower panel) and age-matched non-transgenic mice (non-Tg; upper panel). (B) Arthropathic Tax transgenic mice showed a lower proportion of CD4^+^ T cells and a higher proportion of CD8+ T cells than age-matched non-transgenic mice (n = 8 per group). * *P*<0.05. (C) The ratio of CD4^+^ T cells to CD8^+^ T cells (CD4^+^/CD8^+^) changed from 2.52 in the non-transgenic mice to 0.74 in arthropathic Tax transgenic mice (n = 8 per group) ** *P*<0.01. (D) The expression of *tax* mRNA in the spleen of arthropathic Tax transgenic mice was higher than that in healthy transgenic mice (n = 5 per group) * *P*<0.05.

Flow cytometry using specific monoclonal antibodies against CD19, CD3, CD4 and CD8 markers was used to determine the phenotype of splenocytes from arthropathic Tax transgenic mice. A representative result from the flow cytometric analysis is shown in Figure 6A. No group differences between non-transgenic and arthropathic Tax transgenic mice were found in the proportion of T cells and B cells (Figure 6A, left panel). The splenocytes of arthropathic Tax transgenic mice showed a higher proportion of CD8^+^ T cells than CD4^+^ T cells. In contrast, aged-matched non-transgenic mice had a higher proportion of CD4^+^ T cells than CD8^+^ T cells (Figure 6B). The ratio of CD4^+^ T cells to CD8^+^ T cells was 2.52 in non-transgenic mice and 0.74 in arthropathic Tax transgenic mice (Figure 6C). We then used real-time RT-PCR to measure the expression of *tax* mRNA in the spleen. Splenocytes from arthropathic Tax transgenic mice showed a 40-fold increase in *tax* mRNA expression compared with age-matched healthy transgenic mice (Figure 6D).

### Decreased frequency of forkhead box protein 3 (Foxp3)^+^ Treg cells in arthropathic Tax transgenic mice

**Figure 7:**
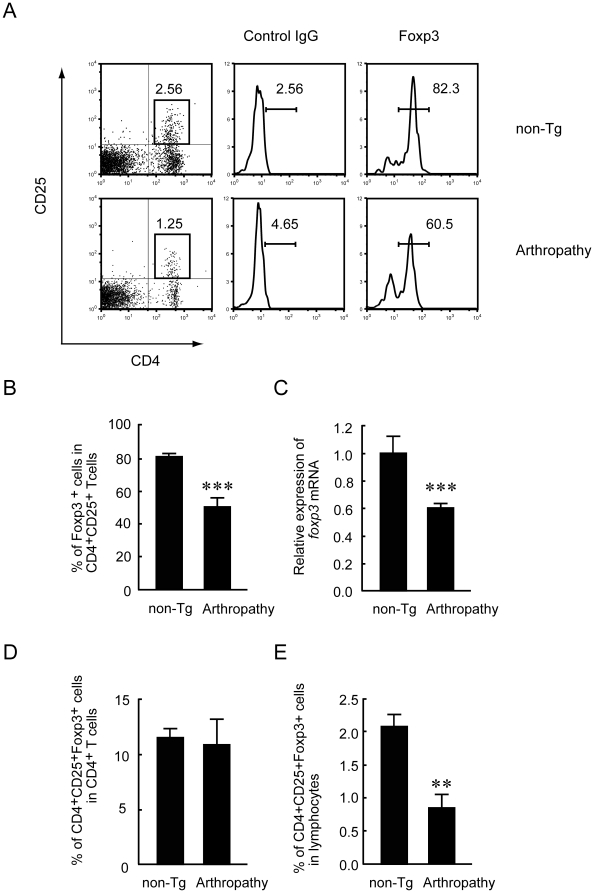
Significantly decreased levels of CD4^+^CD25^+^ Treg cells in arthropathic Tax transgenic mice. (A) Representative flow cytometric analysis of Foxp3 staining in gated CD4^+^CD25^+^ cells in arthropathic Tax transgenic mice (Arthropathy; lower panel) and age-matched non-transgenic mice (non-Tg; upper panel). (B) The majority of CD4^+^CD25^+^ T cells in non-transgenic mice expressed a high level of Foxp3 (81.2±1.4%), whereas half of CD4^+^CD25^+^ T cells in arthropathic Tax transgenic mice did not express Foxp3 (50.2±5.2%) (n = 6 per group), *** *P*<0.001. (C) The relative expression of splenic *foxp3* mRNA was decreased in arthropathic Tax transgenic mice compared with non-transgenic mice (n = 6 per group), *** *P*<0.001). (D) The percentage of CD4^+^CD25^+^Foxp3^+^ Treg cells in CD4^+^ T cells did not differ between arthropathic Tax transgenic mice and non-transgenic mice (n = 6 per group). (E) Arthropathic Tax transgenic mice showed a significant decrease in the percentage of CD4^+^CD25^+^Foxp3^+^ Treg cells in splenic lymphocytes compared with non-transgenic mice (n = 6 per group), ** *P*<0.01.

The proportion of Foxp3^+^ Treg cells is increased in mice with type II collagen–induced arthritis, but Foxp3+ Treg cells do not completely prevent the development of arthritis [17], [29], [30]. We used flow cytometry to determine the proportion of Foxp3^+^ Treg cells in the spleens of arthropathic Tax transgenic mice and non-transgenic littermates. The proportion of CD4^+^CD25^+^ T cells in spleens from arthropathic Tax transgenic mice was slightly lower than that in spleens from non-transgenic mice (Figure 7A). The majority of CD4^+^CD25^+^ T cells in non-transgenic mice expressed a high level of Foxp3 (81.2±1.4%), whereas only about half (50.2±5.2%) of CD4^+^CD25^+^ T cells in arthropathic Tax transgenic mice expressed Foxp3 (Figure 7A, B). Relative expression of *foxp3* mRNA, as determined by real-time RT-PCR, also was lower in the spleens of arthropathic Tax transgenic mice (Figure 7C). The proportion of CD4^+^CD25^+^Foxp3^+^ Treg cells among splenic CD4^+^ T cells was similar among non-transgenic and arthropathic Tax transgenic mice (Figure 7D), probably because of the decrease in splenic CD4^+^ T cells in arthropathic Tax transgenic mice. The proportion of CD4^+^CD25^+^Foxp3^+^ Treg cells among splenic lymphocytes of arthropathic Tax transgenic mice was significantly lower than that of non-transgenic mice (Figure 7E). Patients with HAM/TSP show a similarly lower CD4/CD8 T-cell ratio and proportion of Treg cells [31], [32].

### Increased frequency of CD8^+^CCR4^+^ T cells in arthropathic Tax transgenic mice

**Figure 8:**
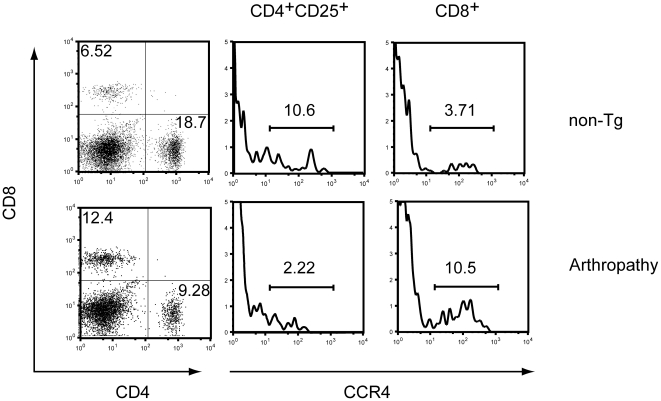
Increased frequency of CD8^+^CCR4^+^ T cells in arthropathic Tax transgenic mice. A representative flow cytometric analysis of CCR4-expressing T cells in gated CD4^+^CD25^+^ or CD8+ T cells in arthropathic Tax transgenic mice (Arthropathy; lower panel) and age-matched non-transgenic mice (non-Tg; upper panel). The proportion of CCR4-expressing CD4^+^CD25^+^ cells in arthropathic Tax transgenic mice (Arthropathy, lower middle panel) was lower than that of age-matched non-transgenic mice (non-Tg; upper middle panel), whereas the percentage of CD8^+^CCR4^+^ T cells was increased in arthropathic Tax transgenic mice compared to non-transgenic mice (right panels).

A recent report showed that a unique proinflammatory T-cell population, IFN-γ^+^CCR4^+^CD4^+^CD25^+^ T cells, termed T_HAM_ cells, is increased in patients with HAM/TSP, with greater proportions observed among patients with more severe disease [33]. We used flow cytometry with a specific monoclonal antibody against CCR4 to determine the proportion of T_HAM_ cells in the spleens of arthropathic Tax transgenic mice and non-transgenic littermates. CCR4-expressing CD4^+^CD25^+^ T cells were rare in all tested arthropathic mice and lower than that seen in non-transgenic mice (Figure 8, middle panel). When CD4^+^CD25^+^CCR4^+^ T cells from both types of mice were cultured, no difference was found in IFN-γ expression (data not shown). In contrast, the proportion of CCR4-expressing CD8^+^ T cells was increased in arthropathic Tax transgenic mice compared with non-transgenic mice (Figure 8, right panel).

## Discussion

A transgenic mouse model of HTLV-1 by using a mouse *Lck* distal promoter to express *tax* in mature thymocytes and peripheral T lymphocytes developed mature T-cell leukemia/lymphoma, similar to ATL [7]. The Tax transgenic mice also develop an inflammatory arthropathy that is pathologically similar to human rheumatoid arthritis and to mouse models of rheumatoid arthritis, with synovial proliferation and rheumatoid factor expression [22], [23], [34]. Transgenic mice carrying the HTLV-1 *env-pX* region (*pX* transgenic mice) or *tax* with the long terminal repeat as a promoter develop inflammatory arthritis at high incidence [8], [9]. Our established Tax transgenic mice differ in several respects from these other transgenic mice. The Tax transgenic mice developed arthropathy after a prolonged latency period of at least 9 months, whereas the arthropathy that develops in *pX* transgenic mice occurs as early as 4 weeks of age. At 3 months of age, 60% (BALB/c background), 25% (C3H/He background), and 0% (C57BL/6 background) of the *pX* transgenic mice are afflicted with arthropathy [35]. The genetic background of our Tax transgenic mice was the F1 generation of a BDF1 (DBA/2 × C57BL/6) crossed with C57BL/6. We attempted to generate Tax transgenic mice using the BALB/c background (backcross generation 8: N8); however, we did not observe a high incidence of arthropathy by 24 months of age (data not shown). Cytokine gene expression of IL-1β, IL-6, and MIF was markedly enhanced in the joints of our Tax transgenic mice, but expression of TNF-α was not increased. Habu *et al.* [36] reported that mRNA expression of IL-1β, IL-6, MIF, and TNF-α in the joints of *pX* transgenic mice was increased compared with those in non-transgenic control mice. In contrast, Ashino *et al.* [37] found that serum IL-1β and IL-6 concentrations in *pX* transgenic mice were significantly higher than those in non-transgenic or non-arthritic *pX* transgenic mice; however, serum TNF-α concentrations were low, with no significant differences between groups. IL-6 is a key proinflammatory cytokine that is abundant in the synovium and synovial tissue of patients with rheumatoid arthritis [38]. Our results suggest that proinflammatory cytokines, except for TNF-α, are important in the development of the inflammatory arthropathy associated with Tax expression.

Collagen is a potent inducer of arthritis in mice and rats [23]. The major histocompatibility complex loci influence the susceptibility of mice to collagen-induced arthritis. Mice with the H-2^q^ (DBA/1) or H-2^r^ (B10.RIII) haplotypes, and their congenic strains, are highly susceptible to collagen-induced arthritis, whereas those with the H-2^b^ (C57BL/6) or H-2^k^ (C3H) haplotypes rarely develop arthritis [24]-[26]. C3H/HeN mice, which have the H-2^k^ haplotype, are low responders to collagen, although *pX* transgenic mice, which have a C3H/HeN genetic background, develop collagen-induced arthritis. Kotani *et al.* [28] suggested that collagen might be an arthritogenic antigen in the joints of *pX* transgenic mice and that oligoclonal T-cell populations with the same TCR Vβ repertoires accumulate in collagen-induced arthritic joints as in arthritic transgenic joints without collagen immunization. Of note, our established Tax transgenic mice, DBA/2 (H-2^d^) × C57BL/6 (H-2^b^), did not develop arthritis after immunization with type II collagen, in contrast to *pX* transgenic mice [27], [28]. Thus, different mechanisms underlie the development of arthropathy in Tax transgenic mice and *pX* transgenic mice, even though both mice express Tax.

The induction of collagen-induced arthritis in susceptible mice involves the activation of helper T cells and B cells [39], [40]. CD4^+^ T cells play an important role in disease progression in the collagen-induced arthritis model, as evidenced by the fact that anti-CD4 treatment suppresses the onset of arthritis [10]. Th1 cells, which produce IFN-γ, are considered the major player in collagen-induced arthritis [41]–[44]. In fact, splenocytes from collagen-induced arthritic mice produce a large amount of IFN-γ after antigen stimulation *in vitro* [12]. More recently, IL-17-producing Th17 cells were identified. Th17 cells are implicated in the pathogenesis of autoimmune diseases based on studies showing that neutralization or genetic deletion of IL-17 inhibits the development of autoimmune diseases in several mouse models, including the collagen-induced arthritis model [14], [45]–[47]. In contrast, arthropathic Tax transgenic mice have a significantly decreased proportion of splenic CD4^+^ T cells. Arthropathic Tax transgenic mice also do not have an increased level of serum IL-17A or proportion of splenic Th17 cells (Figure S1). Furthermore, CD8^+^ T cells are increased in arthropathic Tax transgenic mice. These results suggest that, unlike collagen-induced arthritis, arthropathy in Tax transgenic mice does not involve IL-17A and Th17 but may involve CD8^+^ T cells.

Treg cells are potent suppressors of T-cell responses and can play a critical role in preventing autoimmunity. It was recently demonstrated that Foxp3 is required for Treg differentiation and function [15]. Paradoxically, rheumatoid arthritis develops in humans in the presence of CD4^+^CD25^+^ Treg cells, and Treg cells are enriched at primary sites of autoimmune pathology [48]–[52]. Treg cells are also increased in animal models of rheumatoid arthritis, such as collagen-induced arthritis. The up-regulation of Treg cells may reflect a physiological counter-mechanism to regulate harmful autoimmune responses [53]. In contrast to these findings, arthropathic Tax transgenic mice have a decreased proportion of splenic CD4^+^CD25^+^ T cells and a reduction in Foxp3 expression on splenic CD4^+^CD25^+^ T cells; these results are similar to those observed in HAM/TSP patients [32].

Yamano *et al*. [33] reported that T_HAM_ cells are increased in HTLV-1-infected T cells in HAM/TSP patients, whereas T_HAM_ cells are rare in ATL patients and in healthy individuals. Arthropathic Tax transgenic mice did not have detectable levels of T_HAM_ cells; however, they did have increased CD8^+^ T cells expressing the chemokine receptor CCR4 (CD8^+^CCR4^+^). The proportion of CD8^+^CCR4^+^ T cells is increased in the peripheral blood of patients with psoriasis and palmoplantar pustulosis [54], [55], and CD8^+^CCR4^+^ T cells effectively produce IL-4, IFN-γ, IL-2, and TNF-α [56]. These reports suggest that CD8^+^CCR4^+^ T cells may be involved in the pathogenesis of these diseases. Future studies should examine levels of both CD4^+^CCR4^+^ T cells and CD8^+^CCR4^+^ T cells in HAM/TSP patients.

The bilateral tarsal joint, which supports the whole weight of a mouse, is destroyed in arthropathic Tax transgenic mice. We speculate that blood flow in this joint may be lower than in other joints. Tax expression may reduce the expression of Foxp3, which regulates Treg development and function [15]. Furthermore, age may reduce the suppressor function of Treg cells. Autoimmune T cells, such as CD8^+^CCR4^+^ T cells, may not be suppressed by Treg cells and thus may enter the circulation. Autoimmune T cells may then infiltrate the tarsal joint where the blood flow is lower.

In conclusion, this study provides new and important information on the pathogenic mechanism of arthropathy in Tax transgenic mice, which differs from the pathogenic mechanism of rheumatoid arthritis in other mouse models. Tax transgenic mice develop rheumatoid-like arthritis with proliferating synovial cells in the joints; however, T-cell subsets in these mice resemble those of HAM/TSP rather than rheumatoid arthritis. These results raise the possibility that Tax transgenic mice might be used to study the pathogenic mechanisms of, or therapeutic agents for, HAM/TSP, a disease for which an animal model has not yet been developed.

## Materials and Methods

### Ethics Statement

This study was carried out in strict accordance with the recommendations in the Guidelines for Proper Conduct of Animal Experiments, Science Council of Japan (http://www.scj.go.jp/en/animal/index.html). All procedures involving animals and their care were approved by the Animal Care Committee of Kumamoto University in accordance with Regulations for Animal Experiments in Kumamoto University (approval ID: B22-122).

### Generation of Tax transgenic mice

We developed a transgenic mouse model of HTLV-I using a mouse *Lck* distal promoter to express *tax* in mature thymocytes and peripheral T lymphocytes. A 1.3-kb gene fragment encompassing *tax* was amplified by nested PCR using Ex Taq (Takara Bio, Shiga, Japan) from the *tax* expression vector, pH2R40M [57]. pH2R40M was a gift from Drs. T. Nakashima and M. Nakazawa, St. Marianna University School of Medicine (Kawasaki, Janan). Primers used for the first PCR were MTAX11 and MTAX01 [58]. After the first PCR reaction with 30 cycles, an aliquot of the amplified product was subjected to an additional 30 cycles in a second PCR using internal primers, TAX1 (5′-CTTAATAGCCGCCAGTGGAA-3′) and BTAX0 (5′-CGCGGATCCCGGAGGTCTGAGCTTATGAT-3′); the *Bam*HI site is underlined. Each PCR cycle consisted of denaturation at 94°C for 1 min, annealing at 55°C for 1 min, extension at 72°C for 1.5 min, and extension in the final cycle at 72°C for 10 min. The amplified fragments were digested with *Bam*HI, separated by agarose gel electrophoreses, purified, and subcloned into the *Bam*HI site of the pUC19 vector containing the *Lck* distal promoter, pw120 [59]. pw120 was a gift from Drs. T. Nakayama and C. Shimizu, Chiba University School of Medicine (Chiba, Japan). The subcloned 1.3-kb plasmid DNA in one of the colonies was sequenced and found to match completely with the reported *tax* sequence [58], [60]. After being checked for proper orientation using PCR, the plasmid was digested with *Not*I, and linear insert DNA was prepared (Figure S2A) and microinjected into the pronuclei of BDF1 (C57BL/6 × DBA/2) mouse zygotes. Offspring were obtained after implantation. We biopsied 1-cm portions of tail from 4-week-old mice under ether anesthesia. Tail DNA was isolated using DNA extraction kits (Qiagen, Valencia, CA). To verify integration of *tax* in the host chromosome, tail DNA was digested with *Bam*HI and *Eco*RI and analyzed by Southern blotting using the SK45 probe[61]. Labeling of the probe was carried out by PCR with digoxigenin 11-deoxy UTP (DIG-11-dUTP: Roche Diagnostics, Mannheim, Germany). Specific hybridization was visualized by an enzymatic color reaction on the filter (Figure S2B). A 1.3-kb band of the expected length for *tax* was detected in *Bam*HI-digested genomic DNA from the transgenic mice (Figure S2B, left). A 7.5-kb band of the whole transgene was detected in *Eco*RI-digested genomic DNA. *Eco*RI digests one site in the transgene; thus, the transgene in the transgenic mice was integrated in a head-to-tail configuration. The *tax* copy number was examined by Southern blotting of *Bam*HI-digested genomic DNA from transgenic mice in parallel with a serially diluted plasmid-containing *tax* cDNA. The copy number was estimated to be approximately 20 copies per genome (Figure S2B, right). The expression of *tax* mRNA in various organs of transgenic mice was examined by quantitative real-time RT-PCR. The thymus and spleen strongly expressed *tax* mRNA (Figure S2C).

### Animal maintenance

Tax transgenic mice (background strain BDF1) were crossed with C57BL/6 mice. The F1 mice were used for the present studies. Integration of *tax* into the host chromosome was verified. Genomic DNA prepared from the tail of each mouse using DNA extraction kits (Qiagen). Routine identification of transgenic mice was done by PCR [61], [62]. For Southern blot analysis, genomic DNA (10 µg) was digested with *Bam*HI and *Eco*RI, electrophoresis was conducted using a 0.9% agarose gel, followed by Southern blotting with a probe containing the SK45 sequence [61]. The mice were maintained under specific pathogen-free conditions in laminar-flow benches at 22±2 °C with a 12 h light/dark cycle. Food (CE-2; CLEA, Tokyo, Japan) and water were supplied *ad libitum*. The animals were euthanized by an overdose of sodium pentobarbital anesthesia, and all efforts were made to minimize suffering.

### Real-time PCR

Total RNA from lesion sites of arthropathic Tax transgenic mice and from spleens of age-matched healthy transgenic mice was isolated using RNA extraction kits (Qiagen). Approximately 0.5 µg of total RNA was reverse transcribed with a cDNA Reverse Transcription kit (Applied Biosystems, Foster City, CA). To measure HTLV-1 *tax* expression, real-time PCR was performed as described [62]. Expression of *tax* mRNA in lesions of each disease was calculated and compared to that in spleens of healthy aged-match Tax transgenic mice.

To measure *foxp3* expression in splenocytes of arthropathic Tax transgenic mice, real-time PCR was performed using a SYBR Green PCR Master Mix (Applied Biosystems) according to the manufacturer's instructions on an ABI-Prism 7700 Sequence Detector (Applied Biosystems). Primers for *foxp3* were as follows: *foxp3* sense, 5′-TACACCCAGGAAAGACAGCAACCT-3′, and antisense, 5′-TCTGCTTGGCAGTGCTTGAGAA-3′ [63]. Amplification of mouse *β-actin* (sense, 5′-TGTCCCTGTATGCCTCTGGT-3′, and antisense, 5′-GATGTCACGCACGATTTCC-3′) was used to normalize values of target gene expression.

### Clinical evaluation

Arthropathy was evaluated until 24 months of age. Joint swelling was monitored in blinded cages by two independent animal technicians. Joints of each paw were examined weekly, and scoring was performed as follows: 0  =  normal; 1  =  swelling and redness of the joints; 2  =  obvious swelling; 3  =  joint rigidity.

### X-ray examination

X-ray examination of the joints of arthropathic mice was conducted using a µFX-1000 digital microradiography system (Fujifilm, Tokyo, Japan) set at 25 kV and 80 µA with a 6-sec exposure with an imaging plate. The film was scanned and analyzed using a BAS-2000 bioimage analyzer (Fujifilm).

### Administration of type II collagen

Chicken type II collagen (Chondrex, Redmond, WA) (2 mg/ml) was emulsified 1∶1 with Freund's complete adjuvant (Difco Laboratories, Detroit, MI). Collagen (100 µg) was injected intradermally at the base of the tail of male mice at 2–3 months of age (young) or 15–16 months of age (old). Three weeks after the first immunization, 100 µg of chicken type II collagen emulsified 1∶1 with Freund's incomplete adjuvant was similarly injected.

### Tissue collection and assays

The mice were euthanized with an overdose of sodium pentobarbital, blood samples were obtained at the time of death by direct cardiac puncture. Serum was frozen at −80° C for various assays to be performed at a later date. Spleens were collected, and single-cell suspensions were made for use in flow cytometry. Joints were harvested for tissue histology and analysis of cytokine mRNA. Joints were fixed in 10% neutral-buffered formalin immediately after removal, embedded in paraffin, cut into 4- µm sections, and stained with hematoxylin and eosin using standard protocols.

### Semi-quantitative RT-PCR

Cytokine mRNA expression was measured in the swollen joints of arthropathic mice and the normal joints of healthy mice using semi-quantitative RT-PCR. Briefly, the joints were removed quickly from the skin, frozen in liquid nitrogen, and stored at −80°C. Frozen joints (without the skin) were homogenized with a Polytron homogenizer (PT1300D; Kinematica, Inc., Bohemia, NY). Total RNA was extracted from the joints using an RNA extraction kit (Qiagen) and treated with DNase. Total RNA was then reverse transcribed with reverse transcriptase, and the resultant cDNAs were amplified using primers specific for *IL-1β*, *TNF-α*, *IL-6*, and *MIF* and a mouse *β-actin* primer pair as an internal control [64]. Primers for *IL-1β*, *TNF-α*, *IL-6*, *MIF*, and *β-actin* were as follows: *IL-1β* sense, 5′-CCCAAGCAATACCCAAAGAA-3′, and antisense, 5′-CATCAGAGGCAAGGAGGAAA-3′; *TNF-α* sense, 5′-GCCTCTTCTCATTCCTGCTT-3′, and antisense, 5′-CACTTGGTGGTTTGCTACGA-3′; *IL-6* sense, 5′-TTCCATCCAGTTGCCTTCTT-3′, and antisense, 5′-ATTTCCACGATTTCCCAGAG-3′; *MIF* sense, 5′-TGACTTTTAGCGGCACGAAC -3′, and antisense, 5′-GACTCAAGCGAAGGTGGAAC-3′; and *β-actin* sense, 5′-TGTCCCTGTATGCCTCTGGT-3′, and antisense, 5′-GATGTCACGCACGATTTCC-3′. The PCR was carried out by heating the mixture at 94°C for 2 min followed by 35 cycles of denaturation at 94°C for 30 sec, annealing at 60°C for 30 sec, extension at 72°C for 1 min, and extension in the final cycle at 72°C for 10 min.

### Measurement of cytokine concentrations

Serum concentrations of IL-1β, IL-6, IL-17A, TNF-α, and IFN-γ were determined by qualitative analysis using an ELISA kit for the detection of mouse inflammatory cytokines (SABiosciences, Frederick, MD) and by quantitative analysis using the single cytokine detection kit (R&D Systems, Minneapolis, MN).

### Measurement of NF-κB activity

Nuclear fractions were prepared from joint tissue using a nuclear extraction kit according to the manufacturer's protocol (Active Motif, Carlsbad, CA). DNA-binding activity of NF-κB p65 was then measured using microplate-based ELISA according to the manufacturer's protocol (Active Motif).

### Measurement of Ca^2+^ levels

Serum Ca^2+^ levels were measured by a QuantiChrom calcium assay kit (BioAssay Systems, Hayward, CA) according to the manufacturer's protocol.

### Quantification of autoantibodies

Serum levels of anti-ssDNA IgG and rheumatoid factor were measured using ELISA kits for the detection of mouse anti-ssDNA and IgG rheumatoid factor (Shibayagi, Gunma, Japan).

### Flow cytometry

Cell populations from spleens were examined using flow cytometry. Single-cell suspensions were prepared from the enlarged spleens of transgenic mice and from the spleens of non-transgenic littermates, which were used as controls. The samples were treated with ammonium chloride to lyse red blood cells. For two-color staining, cells were stained with fluorescein isothiocyanate **(**FITC)−labeled anti-mouse CD3 (145-2C11) and phycoerythrin (PE)-labeled anti-mouse CD19 (MB19-1) from eBioscience (San Diego, CA). For three-color staining, cells were stained with FITC-labeled anti-mouse CD4 (RM4-5), PE-labeled anti-mouse CD8 (53-6.7), and allophycocyanin (APC)-labeled anti-mouse CD3 (145-2C11) from eBioscience. To determine the intracellular expression of the Treg cell–specific transcription factor Foxp3 in CD4^+^CD25^+^ cells, mouse splenocytes were first surface-stained with PE-labeled anti-mouse CD4 (RM4-5) and APC-labeled anti-mouse CD25 (PC61.5) from eBioscience. The cells were then fixed using a staining buffer set (BioLegend; San Diego, CA) and intracellularly stained with Alexa Fluor 488−labeled anti-mouse Foxp3 (BioLegend) or Alexa Fluor 488−labeled anti-mouse IgG1,κ (BioLegend). For four-color staining, cells were stained with FITC-labeled anti-mouse CD8 (53-6.7; eBioscience), PE-labeled anti-mouse CCR4 (2G12; BioLegend), peridinin chlorophyll protein (PerCP)−labeled anti-mouse CD25 (PC61; BioLegend), and APC−labeled anti-mouse CD4 (RM4-5; eBioscience). Intracellular cytokines were labeled after activation of lymphocytes by culture for 5 h with phorbol 12-myristate 13-acetate and ionomycin (50 ng/ml and 1 µg/ml, respectively). Splenocytes were then stained using a mouse Th1/Th17 phenotyping kit (BD Biosciences; San Jose, CA). Flow cytometry was conducted using a FACSCalibur (Becton Dickinson; Franklin Lakes, NJ) with FlowJo software (Tree Star; Ashland, OR). Forward and side scatter profiles were used to gate on the lymphocyte population.

### Statistical analysis

All data are presented as the mean ± SEM. Unless otherwise indicated, statistical testing was performed using a two-tailed, unpaired, Student's t-test for the analysis of differences in mean values between groups. We assessed statistical significance for the gender difference with respect to the cumulative incidence of T-cell leukemia/lymphoma or arthropathy in mice with Fisher's exact test. P values<0.05 were considered significant.

## Supporting Information

Figure S1 **Phenotype of Th1/Th17 cells in arthropathic mice.** The staining of interferon-γ (IFN-γ) and IL-17A on resting splenocytes (left panel) and splenocytes stimulated with phorbol 12-myristate 13-acetate/ionomycin (right panel). A small number of IL-17-producing cells was detected in age-matched non-transgenic mice (A), whereas very few IL-17-producing splenocytes were detected in arthropathic Tax transgenic mice (B) (0.5% and <0.1%, respectively). The dot plots are derived from a combined forward- and side-scatter and CD4^+^ gate.
(TIF)

Figure S2 **Construction of the** ***tax*** **transgene and** ***tax*** **mRNA expression in transgenic mice.** (A) Schematic diagram of constructs used for peripheral T cell–specific expression of *tax*. HTLV-1 *tax* was inserted into the *Bam*HI site of the pw120 vector under the control of the mouse *Lck* distal promoter. The 7.5-kb fragment digested by the plasmid *Not*I was used for microinjection. (B) Southern blot analysis of the transgene in Tax transgenic mice (Tg). The position of the *tax* hybridization probe (SK45) is indicated in panel A. The expected bands of 1.3 kb and 7.5 kb were detected in the genomic DNA from Tax transgenic mice digested with *Bam*HI or *Eco*RI (left panel). These bands were absent in non-transgenic mice (N). The *tax* copy number was examined by Southern blotting of *Bam*HI-digested genomic DNA from Tax transgenic mice in parallel with serially diluted plasmid-containing *tax* cDNA (right panel). (C) Relative expression of *tax* mRNA in various organs of Tax transgenic mice was examined using quantitative real-time RT-PCR. *β-actin* was used to normalize the values of target mRNA expression.
(TIF)
