# Supplementary figures and images for: Low CD4/CD8 T-Cell Ratio Associated with Inflammatory Arthropathy in Human T-Cell Leukemia Virus Type I Tax Transgenic Mice
Source: PLoS One. 2011 Apr 1;6(4):e18518. doi: 10.1371/journal.pone.0018518 (PMC3069963; doi:10.1371/journal.pone.0018518)

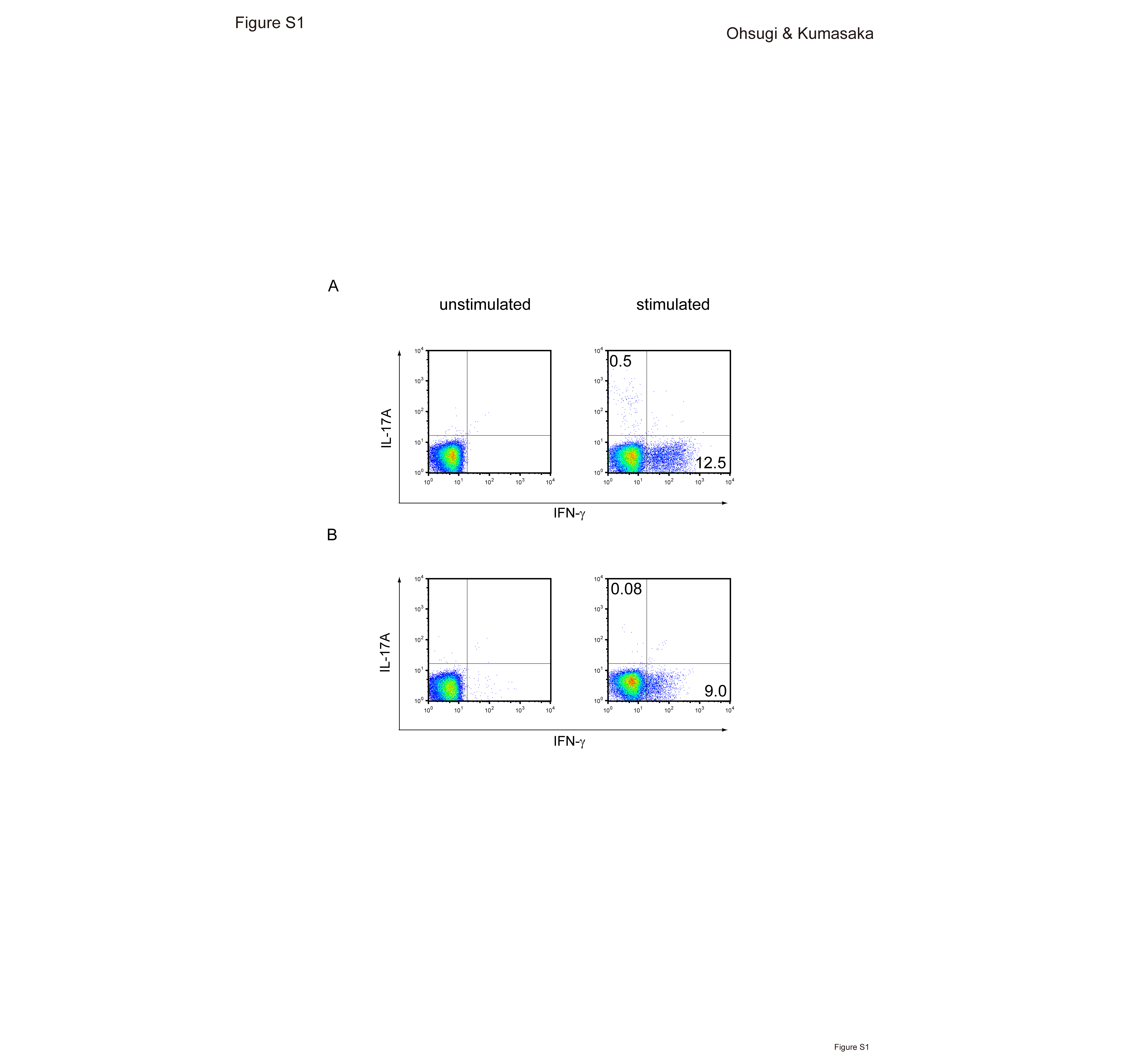

Supplement: Figure S1 — Phenotype of Th1/Th17 cells in arthropathic mice. The staining of interferon-γ (IFN-γ) and IL-17A on resting splenocytes (left panel) and splenocytes stimulated with phorbol 12-myristate 13-acetate/ionomycin (right panel). A small number of IL-17-producing cells was detected in age-matched non-transgenic mice (A), whereas very few IL-17-producing splenocytes were detected in arthropathic Tax transgenic mice (B) (0.5% and <0.1%, respectively). The dot plots are derived from a combined forward- and side-scatter and CD4+ gate. (TIF) [file pone.0018518.s001.tif]

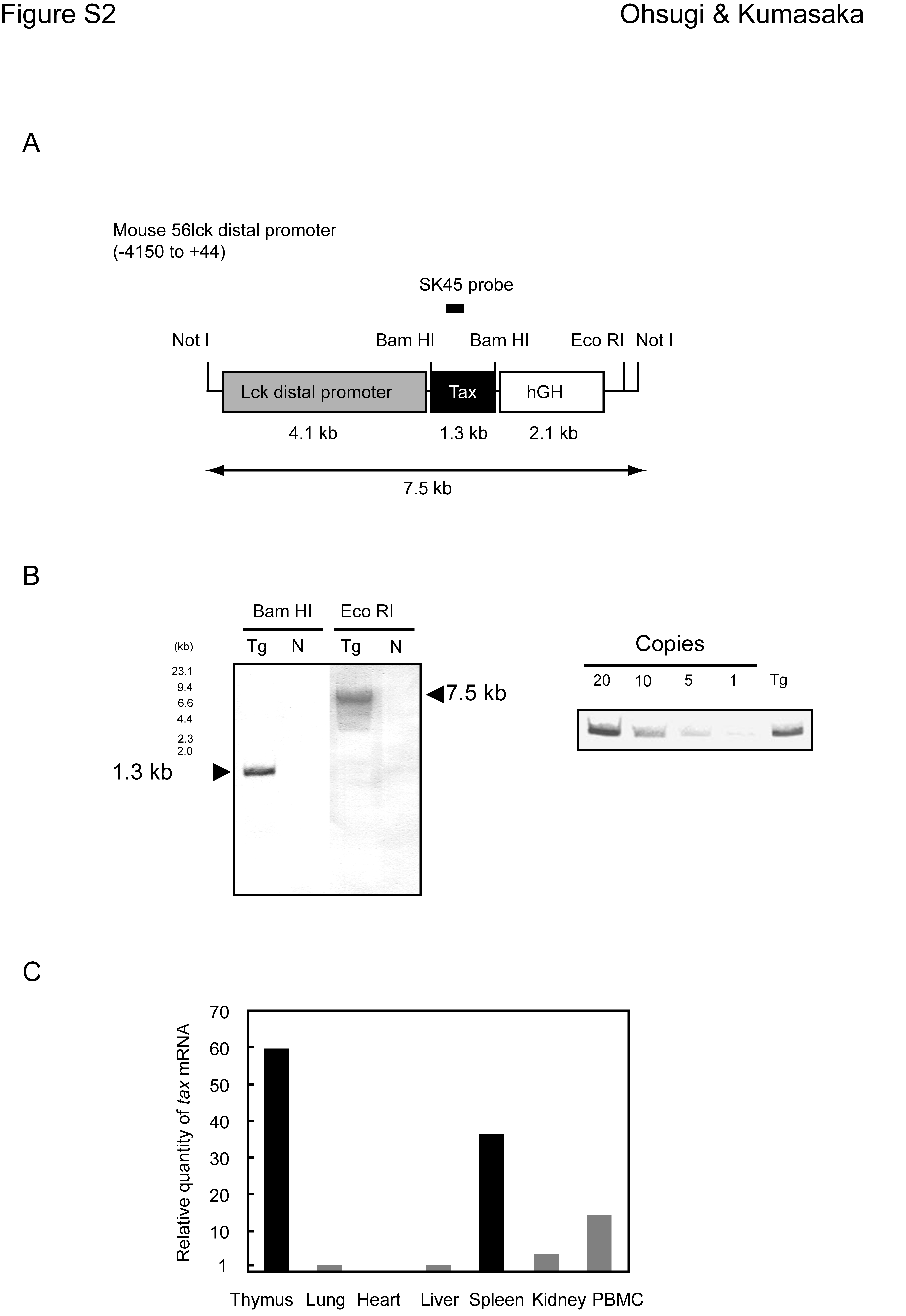

Supplement: Figure S2 — Construction of the tax transgene and tax mRNA expression in transgenic mice. (A) Schematic diagram of constructs used for peripheral T cell–specific expression of tax. HTLV-1 tax was inserted into the BamHI site of the pw120 vector under the control of the mouse Lck distal promoter. The 7.5-kb fragment digested by the plasmid NotI was used for microinjection. (B) Southern blot analysis of the transgene in Tax transgenic mice (Tg). The position of the tax hybridization probe (SK45) is indicated in panel A. The expected bands of 1.3 kb and 7.5 kb were detected in the genomic DNA from Tax transgenic mice digested with BamHI or EcoRI (left panel). These bands were absent in non-transgenic mice (N). The tax copy number was examined by Southern blotting of BamHI-digested genomic DNA from Tax transgenic mice in parallel with serially diluted plasmid-containing tax cDNA (right panel). (C) Relative expression of tax mRNA in various organs of Tax transgenic mice was examined using quantitative real-time RT-PCR. β-actin was used to normalize the values of target mRNA expression. (TIF) [file pone.0018518.s002.tif]
